# Perceptions about the harmfulness of tobacco among adults in Uganda: Findings from the 2013 Global Adult Tobacco Survey

**DOI:** 10.18332/tid/99574

**Published:** 2018-12-20

**Authors:** Steven Ndugwa Kabwama, Daniel Kadobera, Sheila Ndyanabangi

**Affiliations:** 1 Makerere University School of Public Health, Kampala, Uganda; 2 Mental Health and Substance Abuse, Ministry of Health, Kampala, Uganda

**Keywords:** tobacco use, perception, GATS, Sub-Saharan Africa, Uganda

## Abstract

**INTRODUCTION:**

Preferential option for some tobacco products over others might be attributed to inherent misconceptions about the harmfulness of tobacco. We analysed data from Uganda’s Global Adult Tobacco Survey (GATS) to assess misconceptions about the harmfulness of tobacco and associated factors.

**METHODS:**

Data were obtained from the 2013 Uganda Global Adult Tobacco Survey (GATS) of persons in Uganda of age ≥15 years among 8508 participants selected using a multi-stage sampling design to provide nationally representative estimates of the adult population. Participants were asked about perceptions of the harmfulness of smoking, using smokeless tobacco and whether all kinds of cigarettes are equally harmful. Weighted logistic regression analysis was used to find factors associated with the dependent variables.

**RESULTS:**

Among daily smokeless tobacco users, 98 (62%) were unaware that smokeless tobacco causes serious illness. Compared with participants without formal education, participants with primary education were less likely to be unaware that smoking causes serious illness (AOR=0.64, 95% CI: 0.48–0.84) as were participants with secondary education (AOR=0.28, 95% CI: 0.19–0.42) and participants with University education or higher (AOR=0.26, 95% CI: 0.11–0.58). Compared with participants who did not use any smokeless tobacco products, participants who used smokeless tobacco products less than daily were more likely to be unaware that smokeless tobacco causes serious illness (AOR=1.39, 95% CI: 0.54–3.61) as were participants who used smokeless tobacco products daily (AOR=5.87, 95% CI: 3.67–9.40). Compared with participants who did not use any smoked tobacco products, participants who used smoked tobacco products less than daily were more likely to believe that all cigarettes are equally harmful (AOR=2.40, 95% CI: 1.32–4.37) as were participants who used smoked tobacco products daily (AOR=3.08, 95% CI: 2.37–4.00).

**CONCLUSIONS:**

There is a high level of unawareness about the harmfulness of tobacco use particularly among tobacco users. The National Tobacco Control Program should prioritise public awareness and education about the dangers of tobacco use in the Tobacco Control Policy and National Tobacco Control Strategic Plan.

## INTRODUCTION

Research has incontrovertibly established the association between tobacco use and the risk of development of several health effects^1-4^. The World Health Organization (WHO) responded by developing the Framework Convention on Tobacco Control (FCTC)^5^ that emphasises the importance of informing the public of the health consequences, addictive nature and mortality risk from tobacco use and exposure to tobacco smoke. Warning about the dangers of tobacco smoke is also one of the six proven policies of the MPOWER package of the WHO^6^ aimed at reversing the tobacco epidemic. Possessing correct information is critical as it is an indispensable requirement in the accurate perception of risk in a context that can allow for behavioural change^7^. It has also been shown that behavioural change models that focus on changing beliefs about consequences can guide the development of behavioural change interventions^8^.

The most recent nationwide non-communicable risk factor cross-sectional survey carried out in Uganda found that 9.2% of the people in Uganda use tobacco products daily^9^, and yet research elsewhere has shown that tobacco users might preferentially opt for some tobacco products over others because of the false belief that they are safer^10,11^. In 2013, the first Uganda Global Adult Tobacco Survey (GATS) was carried out to ‘establish baseline information on tobacco use and tobacco control measures in a nationally representative sample with regard to exposure to secondhand smoke, cessation, risk perceptions, knowledge and attitudes, exposure to media, price and taxation issues by using a global standard protocol adapted to country-specific context^12^’. We analysed data from this survey to assess the misconceptions about the harmfulness of tobacco and factors associated with it. With the passing of the Uganda Tobacco Control Act 2015^13^, findings from the analysis will provide country-specific data for evaluation of the impact of the legislation in the control of tobacco use. It will also provide valuable information for the development of the Tobacco Control Policy and the National Tobacco Control Strategic Plan.

## METHODS

The data used in this analysis were obtained from the 2013 Uganda Global Adult Tobacco Survey (GATS) of persons in Uganda of age 15 years or older. GATS is a cross-sectional survey that uses a standardised methodology to collect tobacco-related information. Respondents are selected using a multi-stage sampling design that provides estimates that are representative of the country adult population. The sampling design and data collection tools and procedures have been described in detail elsewhere^12^.

### Measures

Smoked tobacco products are those that involve smoking of any part of the tobacco plant while smokeless tobacco products are those that are chewed, inhaled or kept under the gum. Perceptions about the harmfulness of tobacco products were assessed using the following questions: ‘Based on what you know or believe, does smoking cause serious illness?’; ‘Based on what you know or believe, does using smokeless tobacco cause serious illness?’. Categorical response options to both questions were ‘Yes’, ‘No’, or ‘Don’t Know’. Any response other than ‘Yes’ was classified as being unaware of the harmfulness of tobacco use. Perception about the harmfulness of certain tobacco products was assessed with the question: ‘Do you think that some types of cigarettes could be less harmful than other types or are all cigarettes equally harmful?’. Categorical response options were: ‘Could be less harmful’, ‘All equally harmful’, or ‘Don’t know’. Any response other than ‘All equally harmful’ was classified as not knowing that all cigarettes are equally harmful.

### Ethics approval and consent to participate

The survey was conducted by the Uganda Bureau of Statistics (UBOS) on behalf of the Uganda Ministry of Health. The UBOS was formed under the Uganda Bureau of Statistics Act 1998 and is the body with the mandate for collecting, analyzing and publishing national statistics in Uganda^14^. Consent was given by every individual that participated in the survey and all information gathered was kept strictly confidential. The data used for these analyses were publicly available, de-identified data and this research was deemed as being non-human subject research.

### Statistical analysis

Weighted logistic regression analysis was used to find the factors associated with unawareness that smoking causes serious illness, unawareness that smokeless tobacco causes serious illness and unawareness that all types of cigarettes are equally harmful. When calculating the sample weights, data were adjusted for non-response or ineligibility at the household and individual levels. Weights were calculated based on the 2002 Uganda population and housing census^15^ so that the participant sample was nationally representative.

All independent variables suspected to be associated with the dependent variables were put into logistic regression models. The independent variables assessed were gender, age, residence (urban or rural), level of education, marital status, employment status, smokeless tobacco use status and smoked tobacco use status. The independent variables were run in a model against each of the dependent variables. Independent variables were removed one at a time starting with the one least significantly associated with the dependent variable. Variables were significantly associated with the dependent variable if they had a p-value <0.05.

Statistical analyses were performed using STATA version 12. First, the data were declared as being of the complex survey design by using the svyset command. Further analyses were performed using the survey prefix command svy.

## RESULTS

### Study participants

A total of 8982 persons were approached to take part in the survey, of which 8508 (with 16.674 million weighted number of adults) agreed to participate yielding a response rate of 94.9%. There were slightly more females (4655, 54.7%) than males (Table 1). About 7 in 10 participants had some form of employment (5979, 70.3%), majority were married (5013, 59%) and slightly more than half (4382, 51.5%) resided in rural areas. The weighted numbers are included in Table 1.

**Table 1:** Characteristics of study participants of age ≥15 years, GATS 2013, Uganda

*Characteristic*	*Unweighted number of adults N (%)*	Weighted* number of adults (thousands) N
**Total**	8508 (100)	16674
**Gender**		
Male	3853 (45.3)	7870
Female	4655 (54.7)	8804
**Age group (years)**		
15–24	2355 (27.7)	5933
25–44	4230 (49.7)	6869
45–64	1349 (15.9)	2754
≥ 65	574 (6.8)	1117
**Marital status**		
Single	2164 (25.4)	4835
Married	5013 (59.0)	9671
Separated/divorced/	1331 (15.6)	2001
widowed		
**Level of education**		
No formal school	1400 (16.5)	2668
Primary school	4067 (47.8)	8670
Secondary school	2402 (28.3)	4335
University or higher	632 (7.4)	834
**Religion**		
Christianity	7288 (85.7)	14340
Islam	1116 (13.1)	2001
Other	101 (1.2)	167
**Work status**		
Employed	5979 (70.3)	10671
Unemployed	2521 (29.7)	6003
**Residence**		
Urban	4126 (48.5)	4335
Rural	4382 (51.5)	12339
**Relationship with household head**		
Household head	4622 (54.3)	7003
Spouse	2219 (26.08)	4502
Child (son/daughter/grand or step child)	1161 (13.7)	3835
Other	506 (6.0)	1334

* Weighted to the 2002 population and housing census.

Among females, 5.9% were unaware that smoking causes serious illness (Table 2). Among participants older than 65 years, 81 (14.1%) were unaware that smoking causes serious illness. The study also found that among participants who use smokeless tobacco daily, 98 (62%) were unaware that smokeless tobacco causes serious illness and 207 (43.9%) of those who used smoked tobacco daily were unaware that all types of cigarettes are equally harmful.

**Table 2:** Unawareness about harmfulness of different tobacco products among participants of age ≥15 years, GATS 2013, Uganda

*Characteristic*	*n*	*Unaware that smoking causes serious illness*		*Unaware that smokeless tobacco causes serious illness*		*Unaware that all cigarettes are equally harmful*	

		*n (%)*	p*	*n (%)*	*p*	*n (%)*	*p*
**Sex**							
Female	4655	275 (5.9)	0.068	911 (19.6)	0.563	1032 (22.2)	0.174
Male	3853	198 (5.1)		705 (18.3)		895 (23.2)	
**Age group (years)**							
15–24	2355	88 (3.7)	0.000	413 (17.5)	0.000	538 (22.8)	0.000
25–44	4230	204 (4.8)		740 (17.5)		876 (20.7)	
45–64	1349	100 (7.4)		288 (21.3)		325 (24.1)	
≥65	574	81 (14.1)		175 (30.5)		188 (32.8)	
**Residence**							
Urban	4126	199 (4.8)	0.055	738 (17.9)	0.023	876 (21.2)	0.047
Rural	4382	274 (6.3)		878 (20.0)		1051 (24.0)	

* p-value based on chi-squared.

Low level of education, smokeless tobacco use status and smoked tobacco use status were statistically significant predictors of unawareness about the harmfulness of tobacco. Compared with participants with no formal education, participants with primary education were less likely to be unaware that smoking causes serious illness (AOR=0.64, 95% CI: 0.48–0.84) as were participants with secondary education (AOR=0.28, 95% CI: 0.19–0.42) and participants with University education or higher (AOR=0.26, 95% CI: 0.11–0.58) (Table 3). Compared with participants who did not use any smokeless tobacco products, participants who used smokeless tobacco products less than daily were more likely to be unaware that smokeless tobacco causes serious illness (AOR=1.39, 95% CI: 0.54–3.61) as were participants who used smokeless tobacco products daily (AOR=5.87, 95% CI: 3.67–9.40). Compared with participants who did not use any smoked tobacco products, participants who used smoked tobacco products less than daily were more likely to be unaware that all types of cigarettes are equally harmful (AOR=2.40, 95% CI: 1.32–4.37) as were participants who used smoked tobacco products daily (AOR=3.08, 95% CI: 2.37–4.00).

**Table 3:** Crude and adjusted odds ratios (OR) of determinants of being unaware about the harmfulness of tobacco among participants of age ≥15 years, GATS 2013, Uganda

*Characteristic*	*Unaware that smoking causes serious illness*		*Unaware that smokeless tobacco causes serious illness*		*Unaware that all cigarettes are equally harmful*	

	*Crude OR ( 95% CI)*	Adjusted OR* ( 95% CI)	*Crude OR (95% CI)*	Adjusted OR* (95% CI)	*Crude OR (95% CI)*	Adjusted OR** ( 95% CI)
**Sex**						
Female	1.0	1.0	1.0	1.0	1.0	1.0
Male	0.91(0.67–1.22)	0.88(0.67–1.15)	1.05(0.88–1.25)	1.03(0.86–1.22)	1.11(0.95–1.30)	1.10(0.94–1.27)
**Age group (years)**						
15–24	1.0	1.0	1.0	1.0	1.0	1.0
25–44	1.33(0.91–1.94)	1.32(0.92–1.88)	0.91(0.73–1.14)	0.95(0.78–1.16)	0.82(0.68–0.99)	0.78(0.66–0.92)
45–64	1.31(0.81–2.12)	1.33(0.87–2.06)	0.81(0.61–1.08)	0.87(0.67–1.14)	0.74(0.58–0.95)	0.72(0.57–0.91)
≥65	1.60(0.89–2.87)	1.68(0.99–2.83)	1.10(0.75–1.62)	1.24(0.88–1.75)	1.04(0.74–1.46)	1.07(0.79–1.46)
**Level of education**						
No formal school	1.0	1.0	1.0	1.0	1.0	1.0
Primary school	0.75(0.54–1.02)	0.64(0.48–0.84)	0.81(0.65–1.00)	0.78(0.64–0.96)	0.72(0.59–0.88)	0.73(0.59–0.89)
Secondary school	0.35(0.22–0.56)	0.28(0.19–0.42)	0.49(0.37–0.65)	0.47(0.37–0.60)	0.56(0.44–0.72)	0.57(0.45–0.73)
University or higher	0.29(0.13–0.66)	0.26(0.11–0.58)	0.48(0.33–0.70)	0.45(0.31–0.65)	0.63(0.44–0.90)	0.64(0.45–0.90)
**Residence**						
Urban	1.0	1.0	1.0	1.0	1.0	1.0
Rural	1.01(0.79–1.29)	0.99(0.77–1.26)	1.00(0.86–1.15)	0.99(0.86–1.15)	1.06(0.92–1.21)	1.05(0.91–1.21)
**Work status**						
Employed	1.0	1.0	1.0	1.0	1.0	1.0
Unemployed	1.66(1.27–2.18)	1.64(1.25–2.14)	1.30(1.08–1.56)	1.33(1.12–1.58)	0.99(0.83–1.17)	0.99(0.84–1.17)
**Marital status**						
Single	1.0	1.0	1.0	1.0	1.0	1.0
Married	0.93(0.63–1.39)	1.14(0.79–1.64)	1.06(0.84–1.34)	0.99(0.81–1.22)	0.89(0.73–1.09)	0.87(0.72–1.06)
Separated/divorced/widowed	1.14(0.68–1.92)	1.51(0.99–2.30)	1.29(0.92–1.79)	1.21(0.91–1.60)	1.07(0.80–1.44)	1.01(0.77–1.34)
**Smokeless tobacco use status**						
No use	1.0	1.0	1.0	1.0	1.0	1.0
Less than daily	3.61(1.21–10.76)	4.06(1.38–11.95)	1.33(0.52–3.40)	1.39(0.54–3.61)	1.42(0.59–3.42)	1.43(0.60–3.37)
Daily	3.66(2.24–5.98)	3.87(2.37–6.34)	5.80(3.65–9.23)	5.87(3.67–9.40)	4.67(2.91–7.48)	4.70(2.93–7.53)
**Smoked tobacco use status**						
No use	1.0	1.0	1.0	1.0	1.0	1.0
Less than daily	2.10(1.03–4.29)	2.10(1.02–4.33)	1.71(0.92–3.18)	1.74(0.93–3.23)	2.31(1.25–4.28)	2.40(1.32–4.37)
Daily	2.72(1.83–4.04)	2.84(1.99–4.05)	2.50(1.87–3.35)	2.55(1.93–3.37)	2.93(2.24–3.84)	3.08(2.37–4.00)

* Adjusted for level of education, work status, smokeless tobacco use status and smoked tobacco use status.

** Adjusted for age, level of education, smokeless tobacco use status and smoked tobacco use status.

## DISCUSSION

The analysis has revealed that across the three dependent variables, low level of education, smokeless tobacco use status and smoked tobacco use status were statistically significant predictors of unawareness about the harmfulness of tobacco. The fact that more educated people were less likely to be unaware of the harmfulness of tobacco could be that educated people are more likely to comprehend and appreciate anti-tobacco messages and the harmful effects of tobacco use. It could also be that going to school exposes people to more opportunities of acquiring knowledge about the harmful effects of tobacco use. In a worldwide survey of education on tobacco use in schools, it was noted that although 12% of the schools did not cover the topic of tobacco in the curriculum, 58% taught about it while teaching other subjects^16^. Higher education level has also been shown to be a significant predictor of knowledge that smoking causes heart disease, stroke, impotence and lung cancer^17^. Also, persons who attain low levels of education have been shown to smoke more, attempt to quit less, and have a lower likelihood of cessation^18^. This knowledge acquired from educational institutions might play a significant role in correcting any erroneous beliefs but also promoting abstinence. Low education attainment has also been shown to be a significant predictor of adult smoking^19^. In the Ugandan context, education institutions might play a significant role in the fight against tobacco use. In a comparative study of the efficacy of a comprehensive psychosocial smoking prevention program in schools in the US, it was found that students that received this program had better knowledge, personality and life coping skills^20^. In addition to knowledge about the harmful effects of tobacco, schools provide a platform for inculcating other personality and life skills that are important in deterring the initiation of tobacco use.

The survey also established that users of smoked and smokeless tobacco products were significantly more likely to be unaware of the harmfulness of tobacco. Smokers have been shown to have significant gaps in their knowledge of the risks of smoking^21^. Our survey also showed that the unawareness about the harmfulness of tobacco was more pronounced about smokeless tobacco compared with smoked tobacco. For example, 19.6% of females did not believe that smokeless tobacco causes serious illness compared with 5.9% who did not believe that smoking causes serious illness. Research has shown that people perceive smokeless tobacco as more tolerable and less harmful compared with smoked tobacco^22^. One of the documented ways of educating tobacco users on the perils of tobacco use is through instituting graphic health warnings on smokeless and smoked tobacco products^23^. The cross-sectional nature of the survey does not preclude the possibility that the perceptions that people hold about the harmfulness of tobacco might be because of their tobacco use status. The reasons why tobacco users are unaware about the harmfulness of tobacco might be attributed to an optimistic distortion in risk assessment called optimistic bias. Optimistic bias is an error in perception whereby people believe that they are less likely to experience negative events^24^. The bias in judgment occurs in such a way that tobacco users do not believe that they will experience the negative health effects of using tobacco, even though they may be aware of these effects. It has been shown that smokers are more likely to doubt that they would die from smoking if they smoked for 40 years^25^ or even till old age^26^. Optimistic bias has been attributed to the false perception of being in control of the negative events that could happen to an individual^24^. It has been demonstrated that the perception of control could originate from beliefs that the quantity of tobacco someone uses is too little for it to have harmful effects, or that the way someone smokes can protect them from any tobacco use related morbidity^27^. The optimistic distortion in the perception of risk has also been attributed to ineffective warning labels on tobacco product packaging^28^.

Uganda could draw from the practices and strategies that were used to combat the HIV/AIDS epidemic in the early 1990s that involved formalising the information, education and communication about HIV/AIDS^29^. The National Tobacco Control Program could come up with formalised information, education and communication campaigns about the different forms of tobacco, dangers of tobacco use, assistance with quitting and the risks averted with abstinence from use. Also, these campaigns should contain messages that expose the craftiness of the tobacco industry, as this has been shown to be an important ingredient in effective tobacco control programs^30^.

### Strengths and limitations

A major limitation of the survey was that the three dependent variables assessed were based on self-reports, which are a source of information bias. Also, because the study was cross-sectional in design, we cannot be sure that the independent variables were the predictors of the dependent variables and not vice-versa. However, the conduct of the study was systematic enough for the findings to be generalised to the Ugandan population.

## CONCLUSIONS

There is a high level of unawareness about the harmfulness of tobacco use among adults in Uganda. The unawareness is especially high among tobacco users. The National Tobacco Control Program should prioritise public awareness and education about the dangers of tobacco use in the Tobacco Control Policy and National Tobacco Control Strategic Plan.
